# A Case-Based Framework for Teaching Artificial Intelligence Applications in Medical Education

**DOI:** 10.15766/mep_2374-8265.11644

**Published:** 2026-10-27

**Authors:** Rafael C. Schulman, Ali Shervin Razavi, Céline Hardy, Prithi Chakrapani, Abdel Rhman Daoud, Mitchell Mazza, Ankita Saxena, Jason Grullon, Philip J. Gary, Frank Middleton, Sriram Narsipur

**Affiliations:** 1 MD/PhD Student, Department of Biochemistry and Molecular Biology, College of Graduate Studies and Norton College of Medicine, SUNY Upstate Medical University; 2 MD/PhD Student, Department of Neuroscience and Physiology, College of Graduate Studies and Norton College of Medicine, SUNY Upstate Medical University; 3 MD/PhD Student, Department of Microbiology and Immunology, College of Graduate Studies and Norton College of Medicine, SUNY Upstate Medical University; 4 Resident Physician, Department of Surgery, Northwell Health; 5 MD/PhD Student, College of Graduate Studies and Norton College of Medicine, SUNY Upstate Medical University; 6 MD/PhD Student, College of Graduate Studies and Norton College of Medicine, SUNY Upstate Medical University; 7 MD/PhD Student, Department of Neuroscience and Physiology, College of Graduate Studies and Norton College of Medicine, SUNY Upstate Medical University; 8 Resident Physician, Department of Family Medicine, Butler Memorial Hospital – WVU Medicine; 9 Assistant Professor, Department of Medicine, SUNY Upstate Medical University; 10 Professor, Department of Neuroscience and Physiology, SUNY Upstate Medical University; 11 Professor, Department of Medicine, SUNY Upstate Medical University

**Keywords:** artificial intelligence, machine learning, case-based learning, computer vision, pathology, radiology, primary care, ethics/bioethics

## Abstract

**Introduction:**

Artificial intelligence (AI) and machine learning (ML)–based tools are emerging across many areas of medicine. The next generation of medical professionals must be prepared to critically assess the tools they will use, including how they work and the ethical or legal implications of their implementation. To address this need, we developed a generalizable case-writing framework that enables educators and learners to engage with clinical cases integrating AI/ML concepts, clinical reasoning, and ethical considerations.

**Methods:**

We designed a case-writing template to compose clinical case scenarios that integrate clinical, technical, and ethical/legal learning objectives relating to AI applications in medicine. Medical students used this template to construct 3 cases. Student-authored cases were presented to a broader audience in 1-hour sessions: first as a pilot event, then as a class-wide lecture for 140 fourth-year medical students in a transition-into-residency course.

**Results:**

After the sessions, student audiences had a statistically significant increase in familiarity with key AI terminology, a greater appreciation for the importance of ethical considerations of AI applications in health care, and an increased perceived importance of integrating AI-related topics into the medical curriculum.

**Discussion:**

A case-based framework provides a scalable approach to integrating AI education into existing curricula by connecting clinical scenarios with technical concepts and the ethical considerations of AI adoption; cases offer a familiar format that helps learners understand AI applications in patient care while exploring implementation challenges. The case-writing template and 3 sample cases are provided as adaptable resources for educators seeking to incorporate AI literacy.

## Educational Objectives

By the end of this activity, learners will be able to:

1. Explain clinical scenarios in which computer vision algorithms may be used to influence clinical decision-making (eg, vision screening for patients with diabetes, pathology tumor staging)
2. Recognize key ethical and legal considerations of computer vision applications within clinical practice (eg, liability, barriers to access, privacy, and data ownership)
3. Be familiar with core concepts in computer vision and machine learning, including supervised and unsupervised learning, convolutional neural networks, and area under the curve

## Introduction

Artificial intelligence (AI) and machine learning (ML)–based algorithms are poised to have a profound impact on the medical field. At many teaching hospitals and medical universities, AI-based tools are already used across several disciplines,^1^ including radiologic image annotation,^2^ surgery scheduling efficiency,^3^ and patient triage.^4^ As these technological innovations in AI offer new tools in diagnosis and treatment, they also present novel ethical or legal considerations in their application. AI is also being implemented in the teaching environment, with applications including virtual patient simulations,^5,6^ humanoid technology for anatomy education,^7^ personalized learning recommendations,^8^ and generation of assessment questions with large language models.^9^ Despite these rapid advancements, there is little consensus on the most appropriate approach for teaching medical students and trainees about AI.^10^

Therefore, there is a growing urgency for medical curricula to equip students with a foundational understanding of both the technical aspects and the ethical implications of integrating AI into clinical practice. Appropriate education should include fundamental technical information about AI tools while emphasizing the broader clinical context in which they are applied, ensuring students are prepared to use these technologies responsibly in real-world settings.^11^ Moreover, students should be prepared to understand potential ethical and legal considerations and possible pitfalls involved in AI adoption.

The National Academy of Medicine has recommended that health professions schools teach learners responsible AI use.^12^ However, instructional materials to support this goal remain limited. Gordon et al^13^ conducted a comprehensive literature review and identified several effective resources to develop students’ core competencies for critically evaluating AI tools in clinical care. While these resources lay important groundwork, there is still a significant need for educational content that instructors can use to build comprehensive courses on this subject.

To date, 2 *MedEdPORTAL* articles have addressed this gap: Agarwal et al^14^ presented an asynchronous module that provides background on AI and ML to 200 medical students, representing an important step toward meeting the growing need for educational resources in this area. Fernandes et al^15^ presented a case-based ethics session focusing on insurance company medical authorization decisions made by large language model algorithms. Their session applied foundational ethical principles, including autonomy, justice, and beneficence, to a clinically relevant scenario, providing learners with an opportunity to critically examine the ethical implications of AI implementation in health care. They reported improvements in students’ self-assessed understanding of AI-related ethical issues and highlighted the value of peer discussion in facilitating engagement with this emerging topic.

Building on this foundation, there is a need to expand the available resources by continuing to introduce learner-centered approaches that actively engage students in applying AI concepts within realistic clinical scenarios. Like Fernandes et al,^15^ we agree that clinical cases can offer a familiar and effective framework for introducing medical students and trainees to the application of AI in medicine. As the foundational unit of medical education,^16^ cases are central to both preclinical and clinical training—often serving as the primary mode of teaching during clerkships and increasingly integrated into preclinical curricula. Case-based learning is not only preferred by students^17^ but has also been shown to be as effective as, if not more effective than, traditional lecture-based formats.^18^

## Methods

### Case-Based Framework Overview

We developed a case-based instructional framework designed to help medical students critically evaluate AI applications within clinical contexts. This format enables integration of clinical learning points relevant to the medical curriculum with AI-specific technical and ethical learning points.

Our framework consists of 2 separate, complementary components: (1) a structured case development process and (2) presentation of completed cases to learners. The case development process provides an opportunity for deeper engagement by requiring case authors to examine the clinical purpose, technical foundations, and implementation considerations of an AI tool. Subsequent to case development, completed cases were presented to a broader group of learners to explore the same questions and critically evaluate AI applications in clinical practice.

To support case development, we created a structured instructional template for constructing cases about real-world clinical applications of AI. Then, these cases serve as teaching material that can be presented to broader groups of students and trainees. In this article, we share 3 sample student-generated cases focused on computer vision applications in medicine, including examples from radiology, pathology, and retinal imaging (Appendix B). They introduce technical topics, such as model training and neural networks, and discuss ethical issues, such as liability and data ownership. These student-authored cases were presented in a pilot session at a small extracurricular event and in a transition-into-residency session for a full fourth-year medical school class.

### Case-Writing Template and Case Selection

We developed a case-writing template to facilitate case writing (Appendix A). Each case begins with a clinical focus: a patient presentation, followed by brief discussion questions for the audience. These include a possible diagnosis, appropriate management, or pathophysiology of a patient's condition. Each case discusses a patient care challenge and an AI solution developed to address it. Then we introduce technical concepts and vocabulary related to this AI solution. Finally, an ethical, legal, or logistical concern regarding the AI solution is introduced and discussed. Each case concludes by returning to the patient's clinical dilemma and offering a suggested resolution in the context of the AI application.

We developed a library of learning objectives, including technical objectives of core AI/ML concepts and vocabulary, as well as ethical, legal, or logistical considerations. These objectives provided case authors with potential topics to integrate into their respective cases. In parallel, through discussions with colleagues, we considered applications where AI tools were already in use in clinical settings. We also reviewed the US Food and Drug Administration's list of AI/ML-Enabled Medical Devices.^19^ After compiling a list of existing applications, we labeled each application by type: risk stratification, natural language processing, computer vision, or other. To create a single session focused on a single area, we selected 3 applications in a single field for this case series. We chose computer vision, focusing on applications in x-ray image annotation, tumor pathology/histologic grading, and retinal image analysis.

After selecting these 3 applications, we recruited students with backgrounds in ML or engineering to build cases for each application. All 3 authors had prior research experience involving ML, ranging from image analysis and decision tree algorithms to bioinformatics applications and predictive modeling of brain molecular data. Authors were instructed to integrate items from our technical and ethical-legal learning objective lists. We then defined several clinical teaching points for each case to enhance relevance to the general medical curriculum (Table 1). Cases were designed so that each presentation and discussion would occur in an approximate 20-minute time frame.

**Table 1.**
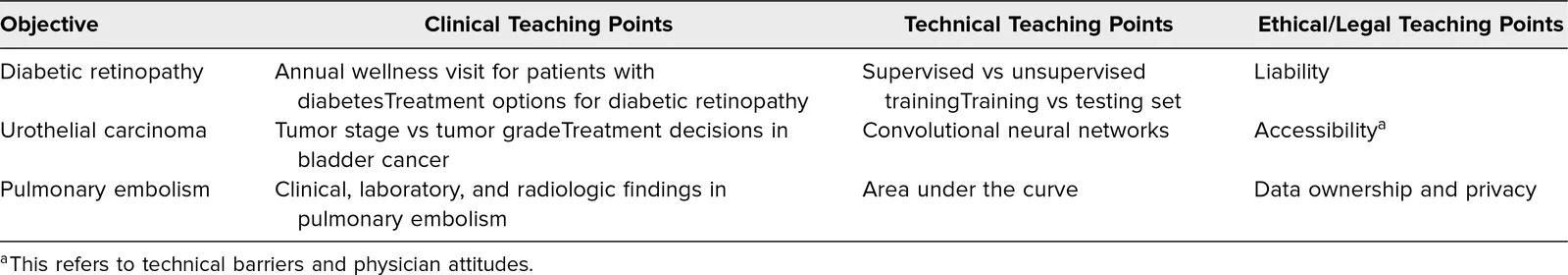
Objectives for Each Clinical Case

### Content Evaluation

We presented these cases in 2 different scenarios. First, as a pilot, cases were presented in an in-person extracurricular event attended by approximately 30 students from all medical school class years. After this pilot, the cases were presented as a component of a transition-into-residency course for graduating fourth-year medical students. The 1-hour session was delivered virtually as part of a daylong program for all graduating students and was attended by 140 students. In both scenarios, the cases were presented as slides, and active engagement was fostered through questions embedded throughout the slides.

Pre- and postsession surveys were administered at both sessions (Appendix C). The surveys were administered by distributing a link and QR code for an online questionnaire, shared in the first and last 5 minutes of the sessions. Surveys were 4 to 8 questions long, including a mix of Likert scale, choose-all-that-apply, and open-ended questions. Surveys were designed to evaluate both attitudes (such as sentiment about AI in health care) and content knowledge (such as familiarity with vocabulary). Surveys were designed to take 3 to 5 minutes to complete, to increase participation and survey completion. Some questions, such as attitudes about whether AI will affect health care careers, were included only in the presession survey. Other questions were included in both the pre- and postsession surveys. This allowed us to evaluate differences in knowledge and attitudes toward AI in medicine after sitting through the sessions. Experience in the pilot session guided modification of the survey for the transition-to-residency session, specifically by adding a terminology familiarity question to the postsession survey.

For the transition-into-residency session, the postsession survey also elicited open-ended feedback from students on topics they want further coverage of, ethical concerns about AI applications in medicine, and any general comments. Responses to these open-ended questions were manually reviewed by the authors to identify common themes and recurring suggestions. Additionally, case authors completed a short survey to share their experience in the case-writing process (included in Appendix C).

This project was reviewed by the SUNY Upstate Institutional Review Board and determined to be exempt (Protocol #2367006-1; determination date October 27, 2025).

## Results

### Pilot Extracurricular Event

A self-selected group of 30 medical students participated in the pilot session, ranging from first to fourth year of medical school, as well as 1 faculty member. Surveys were administered before and after the session and were completed by 16 and 15 participants, respectively. All 16 presession respondents reported an optimistic overall sentiment toward AI's effect on health care. Thirteen (81%) felt that AI would affect their career in medicine. Respondents felt that AI education in medicine was important: 14 of 16 respondents said that a basic level of AI literacy should be a part of medical education.

The session increased students’ perceived importance of education about ethical considerations of AI in medicine. One question asked, “What components do you think should be included in medical education about AI?” Prior to the session, “Ethics of developing new AI tools” was selected by 9 (56.2%) students, which rose to 14 (93%) postsession (Fisher's *P* = .037, Figure). Another ethics question (“Ethics of using existing AI tools”) was not significant (Fisher's *P* = .22) but was selected by 10 (63%) students before the session, which rose to 13 (87%) postsession. Interestingly, all 16 respondents chose “Clinical best practices” in the presession survey, which dropped to 13 of 15 postsession (87%). However, given the relatively small sample size, this difference was not statistically significant by Fisher's exact test (*P* = .226).

**Figure.**
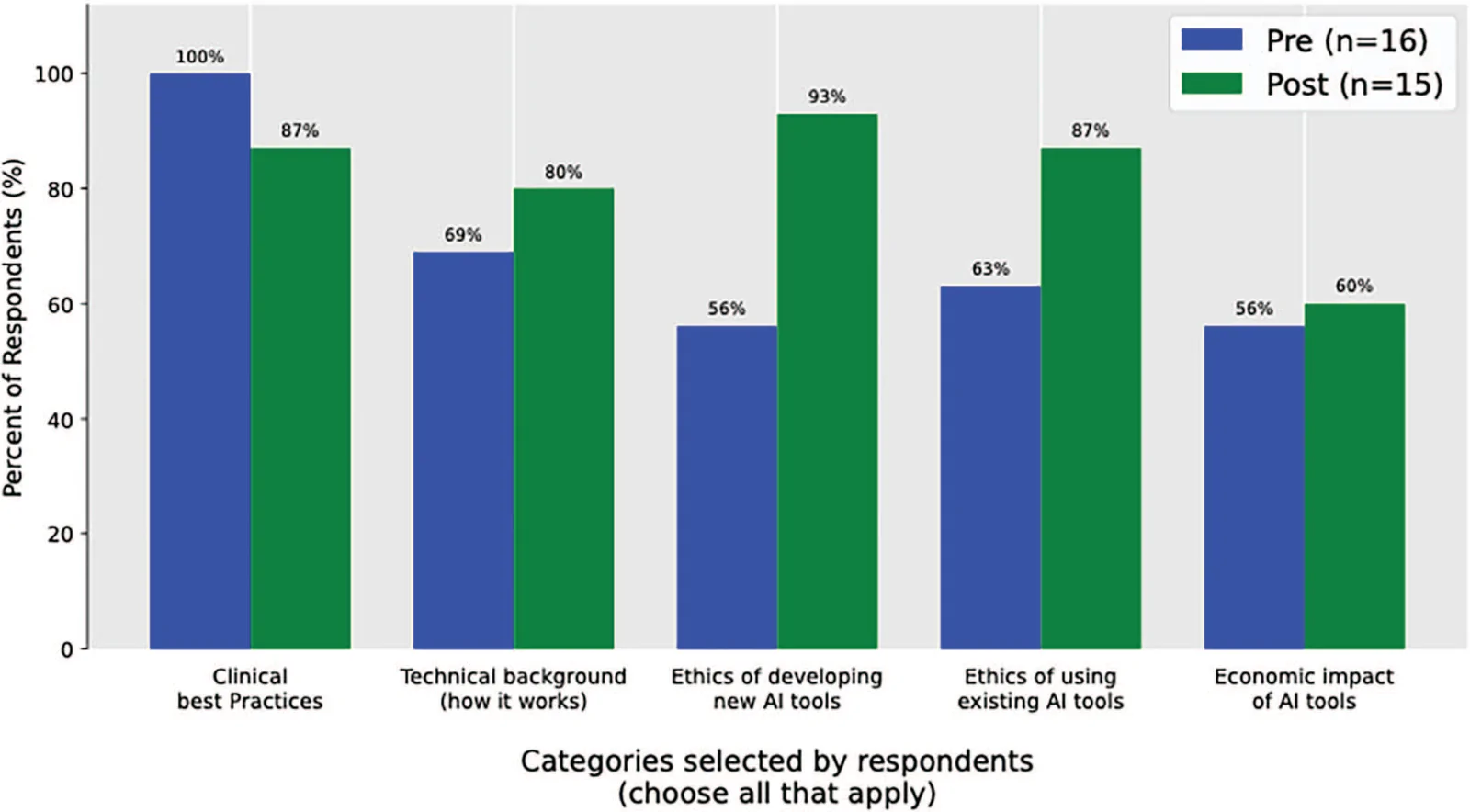
Students’ opinions on the most important components of artificial intelligence (AI) to integrate into medical curriculum before and after the session. Students were asked what topic areas should be included in medical education about AI, in a choose-all-that-apply format. Bar plot indicates the proportion of students/session attendees whose survey responses indicated that they believe a particular component should be incorporated into the medical curriculum, both presession (blue bar, n = 16) and postsession (green bar, n = 15). Components focusing on ethical components of AI, including development of new tools and use of existing tools, had the largest increase in students’ perceived importance for medical curriculum.

### Transition-Into-Residency Course

A total of 140 fourth-year medical students attended this mandatory 1-hour session, presented as a component of the transition-into-residency course. Of the students, 101 (72%) completed the presession survey, and 81 (57%) completed the postsession survey. Given that survey respondents included a full medical school class, responses captured a broader array of attitudes toward AI in medicine, including more skepticism than the extracurricular event; 46.5% reported their sentiment as optimistic, 37.6% reported their sentiment as neutral, and the remaining 15.9% reported their sentiment as pessimistic. Most respondents (66%) thought that AI would affect their career, although it was less than for the extracurricular event (81%).

Prior to the session, students were also more hesitant regarding the importance of AI in the medical school curriculum. The mean score on a scale of 1 to 5 from unimportant to important was 3.73, compared with a mean of 4.44 in the extracurricular event (Mann-Whitney *U* test, *P* < .0001). Only 20.8% of students rated it 5 of 5, compared with 62.5% of the extracurricular event's participants. Notably, this attitude shifted after the session; in the postsession survey, 42% of respondents scored the importance of incorporating AI into medical education as a 5 of 5, and the mean score on a scale of 1 to 5, from unimportant to important, rose from 3.73 to 4.19 (Mann-Whitney *U* test, *P* < .001). Regarding what specific subjects within AI are most important to teach in an AI curriculum, the 2 ethics components, developing new AI tools and using existing AI tools, saw the greatest increase, 10% and 6%, respectively.

### Familiarity With AI/ML Terminology

Familiarity with AI/ML terminology was assessed using a single check-all-that-apply survey item: “Which of the following terms are you familiar with within the context of Artificial Intelligence or Machine Learning?” Respondents were presented with a fixed list of 12 terms—sensitivity and specificity, linear regression, multilayer perceptron, neural network, convolutional neural network, supervised and unsupervised learning, backpropagation, transformers, attention, cross-fold validation, training and testing sets, and large language model—and could select any number of terms they recognized. For the pilot session, this question was administered in the presession survey. To better evaluate the effectiveness of this educational activity for the class-wide transition-into-residency session, this question was administered in both the pre- and postsession surveys (Table 2).

**Table 2.**
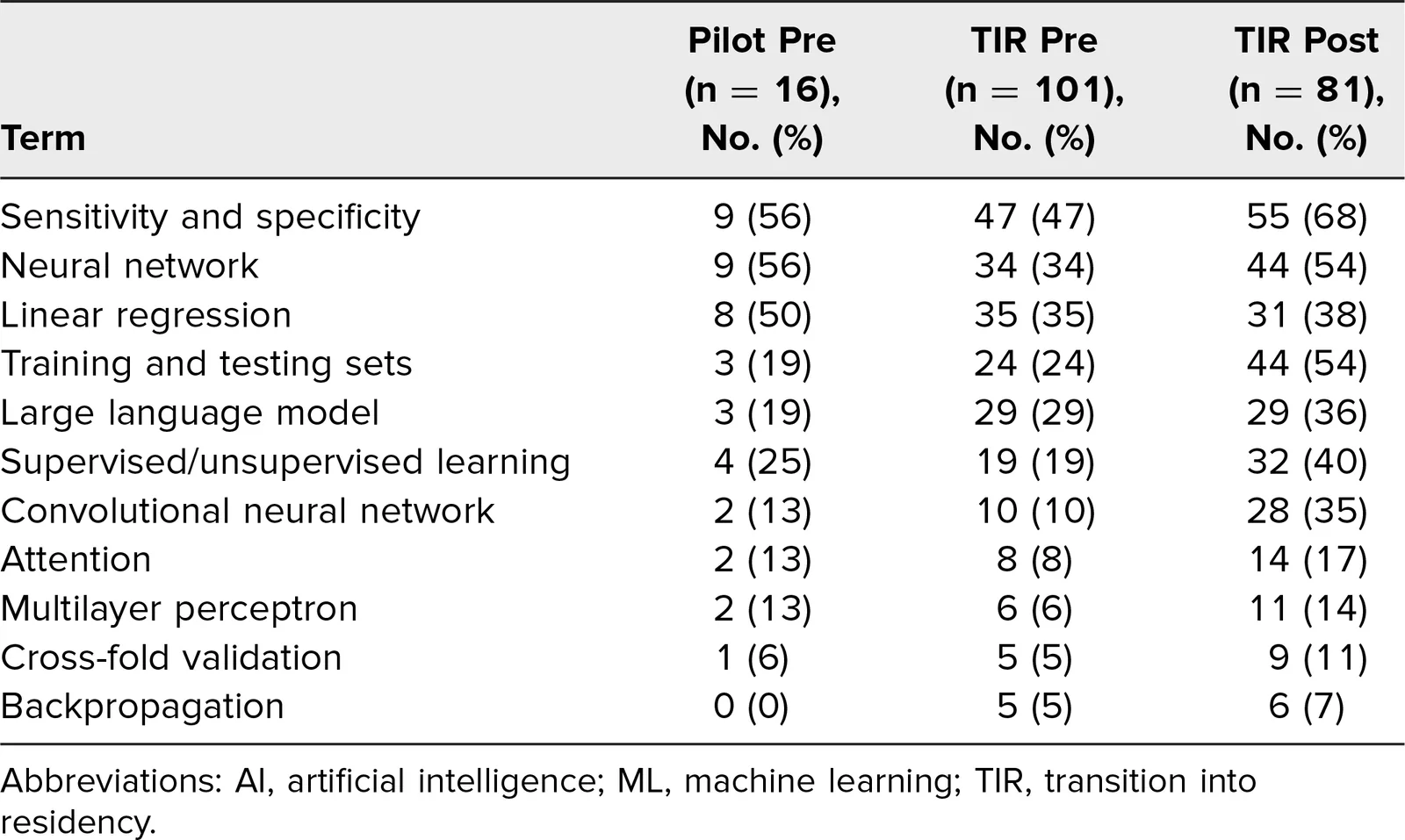
Familiarity With AI/ML Terminology Assessed With Check-All-That-Apply Survey Item in Pilot and TIR Sessions

Presession familiarity was higher among pilot respondents than among transition-into-residency presession respondents on 9 of 12 terminology items (eg, neural network: 56% vs 34%; sensitivity and specificity: 56% vs 47%). A larger proportion of extracurricular respondents reported familiarity with at least 1 term overall (81.3% vs 60.4%), consistent with this group's generally higher baseline engagement observed across other survey items. In the transition-into-residency session, reported familiarity with AI/ML terminology increased markedly following the educational session, with gains concentrated specifically in the concepts the session addressed. Among the 5 terms directly covered in the session (supervised and unsupervised learning, training and testing sets, convolutional neural network, neural network, and sensitivity and specificity), reported familiarity rose by an average of 23.9 percentage points (range, 20.9 to 30.8 points), with every covered term showing a gain exceeding 20 points. In contrast, the 7 terms not covered in the session (linear regression, multilayer perceptron, backpropagation, transformers, attention, cross-fold validation, and large language model) showed much smaller average gains of 5.6 percentage points (range: 2.0 to 9.4 points). This difference in mean gain between terms covered and terms not covered in the session was statistically significant (Mann-Whitney *U* test = 35.0, *P* = .0025). Notably, postsession transition-into-residency familiarity with covered terms approached or exceeded the presession baseline of the pilot extracurricular cohort on 4 of 5 terms (all but neural network, which reached 54.3% vs the extracurricular baseline of 56.2%), despite transition-into-residency participants starting from a lower baseline.

### Student Feedback

After the transition-into-residency session, 14 students responded regarding topics they would want to learn more about, specifying content covering specialty-specific impacts, legal liability, and practical current-use cases like AI scribes and note-writing tools. Regarding ethical concerns, 30 students responded, with concerns centered on liability and accountability for AI errors, patient data privacy and Health Insurance Portability and Accountability Act compliance, and bias in training data and outputs. Cost and overreliance on AI were also recurring secondary themes. Nineteen students shared general closing comments, largely positive, praising the case-based teaching format and pathology coverage, though 2 respondents offered constructive critiques. One felt the content was better suited to earlier training years (MS1/MS2) than residency transition, and another suggested slide design changes to keep patient context visible throughout the slides.

### Case Author Reflections

Three student authors contributed original AI in Medicine cases and provided feedback on their case-writing experience. Case authors reported that the writing process deepened their understanding of domain-specific AI tools, including convolutional neural networks and regulatory distinctions between diagnostic hardware and analytical software. They also reported gaining greater insight into ethical considerations and commercial implementations of AI in clinical settings. All authors found the case-writing template (Appendix A) helpful for organizing and presenting their content. Suggestions for improvement included adding guidance on citation formatting, providing clear style standards (eg, fonts, sizes, colors), and developing a shared document for collaborative learning during case development.

## Discussion

This case-based framework is a practical response to a national need in medical education. Our clinical case-based format fostered increased appreciation and interest in the impact of AI and ML technologies on health care. Students engaged well with the active learning elements of the cases and found value in anchoring the material in patient-centered scenarios that align with their broader clinical training. Student attitudes changed regarding the ethical dimensions of both AI development and deployment. Additionally, students expressed a stronger belief in the importance of AI education following participation in the sessions. These findings suggest that even brief educational interventions can shift perceptions toward supporting AI literacy in medical training and reinforce the importance of ethical awareness among future physicians.

### Case Writing: Opportunities and Limitations

The case-writing process itself provides a nuanced lens for studying how AI is being applied in health care. For instance, as authors of this case series and article, the process of building case-based content toward AI applications in medicine helped us appreciate a key distinction among the tools we explored: screening models, such as those used in diabetic retinopathy detection, are designed to make referral recommendations without producing outputs intended for clinical interpretation. This contrasts with clinical decision support tools, such as algorithms for tumor grading, which assist clinicians through image annotation intended to aid in interpretation. Viewing these technologies through a case-based lens made this distinction more tangible to us and emphasized how context shapes the design and intended use of AI systems in clinical care.

Nevertheless, the case-writing process is inherently constrained by the students or trainees recruited to write cases. The 3 cases reported here were written by students with backgrounds in mathematics, computer science, or engineering, enabling them to integrate technical knowledge with clinical need addressed in each given application. For educators implementing this resource within their curriculum, recruiting medical students with backgrounds in relevant technical fields may be ideal; student-led case writing will be influenced by case writers’ backgrounds and limited by their previous knowledge and ability to research and integrate information when writing cases. Nevertheless, this allows this format to benefit from the strengths of case writers. For instance, if a student has a background in philosophy or ethics, they may be better equipped to explore topics of privacy, bias, or liability. The case-writing process provides a rich and textured way to think critically about AI in health care, necessarily set against the backdrop of case writers’ academic and intellectual background.

### Case-Based Sessions: Shifts in Ethical Attitudes and Terminology Familiarity

After case development, cases were presented to broader audiences in both a pilot extracurricular and larger, required course settings, and survey data captured measurable shifts in attendees’ ethical attitudes and AI/ML terminology familiarity. In the pilot session, pre- and postsession surveys measured a significant shift in attitudes toward the importance of ethics education in AI and health care. Prior to the session, the component of AI education that students rated most important to the medical curriculum was “Clinical best practices.” The session then presented concrete examples of the ethical and legal issues that emerge as AI tools are developed and deployed in clinical settings, including liability when AI augments clinical decision-making and questions of ownership over patients’ private medical data. After the session, “Ethics of developing new AI tools” became the most selected component, with “Ethics of using existing AI tools” and “Clinical best practices” tied for second. This shift suggests that grounding ethical content in concrete, case-based examples that reflect what students will see in clinical settings helps demonstrate the ethical and legal dimensions of tools they will soon encounter in clinical practice. Introducing these considerations earlier in training, within a structured classroom setting, may give students a more complete opportunity to grapple with these issues before facing them under the time pressure of real clinical decision-making.

Since this pilot was a voluntary, extracurricular event, respondents were likely students with a preexisting interest in AI in medicine, reflected in their higher presession familiarity with key AI/ML terminology compared with the transition-into-residency cohort, which captured a full medical school class. To more rigorously assess the educational impact of the transition-into-residency session, we assessed terminology familiarity in both the pre- and postsession surveys, distinguishing terms directly covered in the session from terms not covered, which served as a negative control. Reported familiarity with covered terms rose by an average of 23.9 percentage points, compared with only 5.6 points for uncovered terms. This significant difference (Mann-Whitney *U* test = 35.0, *P* = .001) indicates the gains reflect the session's specific content rather than general survey exposure or retesting effects. Notably, postsession familiarity with covered terms in this cohort approached or exceeded the pilot cohort's presession baseline on 4 of 5 terms, suggesting that a single brief session can close much of the gap between students with and without prior self-selected interest in AI.

### The Need for Validated Measures and Consensus Learning Objectives

During our pilot session, pre- and postsession surveys measured a statistically significant change in attitude toward the importance of ethical issues in AI in health care. Additionally, in the transition-to-residency session, we found that student familiarity with technical terminology showed statistically significant improvement from the presession to the postsession survey. Although this limited assessment demonstrated the effectiveness of our educational activity, the field would benefit from more extensively validated measures to assess student knowledge on subjects related to technical components most relevant to medicine, as well as ethical issues of AI implementation in health care.

Moreover, the medical education community does not currently have a consensus regarding what learning objectives should be prioritized for medical students. There is a need for a clear set of learning objectives for medical trainees who will increasingly be using AI-based tools. In our work, we prioritized ethical considerations of privacy, liability, and access, as well as technical concepts related to performance, model training, and model architecture. Although a case-based approach has provided an effective framework for teaching these technical and ethical objectives, the medical education community still needs to define which learning objectives are most appropriate for medical students, residents, and health care practitioners.

As AI continues to permeate clinical practice, the importance of producing critically engaged and ethically literate providers becomes increasingly urgent. Students must understand not only how AI tools function but also how to evaluate their limitations, risks, and implications for patient care. Our results underscore the value of a case-based approach for integrating ethical reasoning alongside technical content in AI in medicine curricula, promoting relatable engagement for medical trainees. In future work, this case-based framework can be expanded to include cases on natural language processing, risk stratification, and other applications. It can also be tailored to teach specific objectives determined by the medical education community as consensus emerges on which learning objectives are most important. In addition to building foundational knowledge, we hope this model fosters curiosity and involvement among medical trainees, encouraging thoughtful innovation and the responsible integration of AI in medicine.

## Appendices


AI4Med Case Template.pptxAI4Med Computer Vision Cases.pptxSurvey Instruments.docx
*All appendices are peer reviewed as integral parts of the Original Publication.*
