# Supplementary material for: A Case-Based Framework for Teaching Artificial Intelligence Applications in Medical Education
Source: MedEdPORTAL. 2026 Oct 27;22:11644. doi: 10.15766/mep_2374-8265.11644 (PMC13711041; doi:10.15766/mep_2374-8265.11644)
Supplement: Supplementary file 1 [file mep_2374-8265.11644-s001.zip › C. Survey Instruments.docx]

Appendix C. Survey Instruments

*This appendix reproduces the questions, instructions, and response options used in each survey referenced in the manuscript, to support replication of the pre- and post-session audience surveys and the case-author reflection survey. All surveys were administered anonymously and electronically; no identifying information was collected.*

# 1. Extracurricular Event — Pre-Session Survey

Administered immediately before the extracurricular AI-in-medicine session, prior to any instructional content (n = 16 respondents).

**1. What program and year are you?**

*Response format: Open-ended (free text).*

**2. Overall, what is your sentiment about how A.I. will affect healthcare?**

*Response format: 5-point Likert scale (1 = pessimistic, 5 = optimistic).*

**3. Do you think your career in medicine will be affected by A.I. technology?**

*Response format: 5-point Likert scale (1 = no, 5 = very much so).*

**4. Have you ever worried about how A.I. will affect your career in medicine?**

*Response format: 5-point Likert scale (1 = never, 5 = often).*

**5. Do you think a basic level of A.I. literacy should be a part of medical education?**

*Response format: 5-point Likert scale (1 = no, 5 = yes).*

**6. What components do you think should be included in medical education about A.I.?**

*Options (select all that apply): Technical background (how it works); Ethics of using existing AI tools; Ethics of developing new AI tools; Clinical best-practices; Economic impact of AI tools*

**7. What ethical concerns do you think are most important in regard to AI integration in medical care?**

*Response format: Open-ended (free text).*

**8. Which of the following terms are you familiar with within the context of Artificial Intelligence or Machine Learning?**

*Options (select all that apply): Sensitivity and Specificity; Linear Regression; Multilayer Perceptron; Neural Network; Convolutional Neural Network; Supervised and Unsupervised Learning; Back Propagation; Transformers; Attention; Cross-fold Validation; Training and Testing Sets; Large Language Model*

# 2. Extracurricular Event — Post-Session Survey

Administered immediately following the extracurricular AI-in-medicine session (n = 15 respondents).

**1. Do you think a basic level of A.I. literacy should be a part of medical education?**

*Response format: 5-point Likert scale (1 = no, 5 = yes).*

**2. What components do you think should be included in medical education about A.I.?**

*Options (select all that apply): Technical background (how it works); Ethics of using existing AI tools; Ethics of developing new AI tools; Clinical best-practices; Economic impact of AI tools*

**3. Are there any specific topics or subject areas related to AI in medicine that you think are important to cover?**

*Response format: Open-ended (free text).*

**4. Any closing comments?**

*Response format: Open-ended (free text).*

# 3. Transition Into Residency (TIR) — Pre-Session Survey

Administered to fourth-year medical students immediately before the mandatory one-hour AI-in-medicine session held as part of the Transition-Into-Residency course (101 of 140 students, 72%).

**1. Overall, what is your sentiment about how A.I. will affect healthcare?**

*Response format: 5-point Likert scale (1 = pessimistic, 5 = optimistic).*

**2. Do you think your career in medicine will be affected by A.I. technology?**

*Response format: 5-point Likert scale (1 = no, 5 = very much so).*

**3. Have you ever worried about how A.I. will affect your career in medicine?**

*Response format: 5-point Likert scale (1 = never, 5 = often).*

**4. Do you think a basic level of A.I. literacy should be a part of medical education?**

*Response format: 5-point Likert scale (1 = no, 5 = yes).*

**5. What components do you think should be included in medical education about A.I.?**

*Options (select all that apply): Technical background (how it works); Ethics of using existing AI tools; Ethics of developing new AI tools; Clinical best-practices; Economic impact of AI tools*

**6. What ethical concerns in AI, if any, do you think are most important in regard to AI integration in medical care?**

*Response format: Open-ended (free text).*

**7. Which of the following terms are you familiar with within the context of Artificial Intelligence or Machine Learning?**

*Options (select all that apply): Sensitivity and Specificity; Linear Regression; Multilayer Perceptron; Neural Network; Convolutional Neural Network; Supervised and Unsupervised Learning; Back Propagation; Transformers; Attention; Cross-fold Validation; Training and Testing Sets; Large Language Model*

# 4. Transition Into Residency (TIR) — Post-Session Survey

Administered immediately following the TIR session (81 of 140 students, 57%).

**1. Do you think a basic level of A.I. literacy should be a part of medical education?**

*Response format: 5-point Likert scale (1 = no, 5 = yes).*

**2. What components do you think should be included in medical education about A.I.?**

*Options (select all that apply): Technical background (how it works); Ethics of using existing AI tools; Ethics of developing new AI tools; Clinical best-practices; Economic impact of AI tools*

**3. What ethical concerns do you think are most important in regard to AI integration in medical care?**

*Response format: Open-ended (free text).*

**4. Which of the following terms are you familiar with within the context of Artificial Intelligence or Machine Learning?**

*Options (select all that apply): Sensitivity and Specificity; Linear Regression; Multilayer Perceptron; Neural Network; Convolutional Neural Network; Supervised and Unsupervised Learning; Back Propagation; Transformers; Attention; Cross-fold Validation; Training and Testing Sets; Large Language Model*

**5. Are there any specific topics or subject areas related to AI in medicine that you think are important to cover?**

*Response format: Open-ended (free text).*

**6. Any closing comments?**

*Response format: Open-ended (free text).*

# 5. Case Author Survey

Completed by the medical students and trainees who authored the AI-in-medicine teaching cases (Pathology, Diabetic Retinopathy, and Radiology), reflecting on their experience developing case content and using the case-writing template.

**1. Which case did you write?**

*Response format: Open-ended (free text; e.g., Pathology, Diabetic Retinopathy, Radiology).*

**2. What is your background in Machine Learning or Artificial Intelligence?**

*Response format: Open-ended (free text).*

**3. Did you learn anything new when doing background research for your case?**

*Response format: Open-ended (free text; yes/no with explanation).*

**4. Did you find the case-writing PowerPoint template helpful?**

*Response format: Open-ended (free text; yes/no with explanation).*

**5. Do you have any suggestions for improving the case-writing template?**

*Response format: Open-ended (free text).*
